# Air quality index variation before and after the onset of COVID-19 pandemic: a comprehensive study on 87 capital, industrial and polluted cities of the world

**DOI:** 10.1186/s12302-021-00575-y

**Published:** 2021-12-05

**Authors:** Mohammad Sarmadi, Sajjad Rahimi, Mina Rezaei, Daryoush Sanaei, Mostafa Dianatinasab

**Affiliations:** 1 Department of Environmental Health Engineering, School of Health, Torbat Heydariyeh University of Medical Sciences, Torbat Heydariyeh, Iran; 2 Health Sciences Research Center, Torbat Heydariyeh University of Medical Sciences, Torbat Heydariyeh, Iran; 3 Department of Environmental Health Engineering, Faculty of Public Health and Safety, Shahid Beheshti University of Medical Science, Tehran, Iran; 4 Department of Complex Genetics and Epidemiology, School of Nutrition and Translational Research in Metabolism, Maastricht University, Maastricht, The Netherlands

**Keywords:** COVID-19, Air quality index, Lockdown, Restriction, Nitrogen dioxide, Particulate matter

## Abstract

**Background:**

Coronavirus disease 2019 (COVID-19) pandemic provided an opportunity for the environment to reduce ambient pollution despite the economic, social and health disruption to the world. The purpose of this study was to investigate the changes in the air quality indexes (AQI) in industrial, densely populated and capital cities in different countries of the world before and after 2020. In this ecological study, we used AQI obtained from the free available databases such as the World Air Quality Index (WAQI). Bivariate correlation analysis was used to explore the correlations between meteorological and AQI variables. Mean differences (standard deviation: SD) of AQI parameters of different years were tested using paired-sample *t*-test or Wilcoxon signed-rank test as appropriate. Multivariable linear regression analysis was conducted to recognize meteorological variables affecting the AQI parameters.

**Results:**

AQI-PM_2.5_, AQI-PM_10_ and AQI-NO_2_ changes were significantly higher before and after 2020, simultaneously with COVID-19 restrictions in different cities of the world. The overall changes of AQI-PM_2.5_, AQI-PM_10_ and AQI-NO_2_ in 2020 were – 7.36%, – 17.52% and – 20.54% compared to 2019. On the other hand, these results became reversed in 2021 (+ 4.25%, + 9.08% and + 7.48%). In general, the temperature and relative humidity were inversely correlated with AQI-PM_2.5_, AQI-PM_10_ and AQI-NO_2_. Also, after adjusting for other meteorological factors, the relative humidity was inversely associated with AQI-PM_2.5_, AQI-PM_10_ and AQI-NO_2_ (β = − 1.55, β = − 0.88 and β = − 0.10, *P* < 0.01, respectively).

**Conclusions:**

The results indicated that air quality generally improved for all pollutants except carbon monoxide and ozone in 2020; however, changes in 2021 have been reversed, which may be due to the reduction of some countries’ restrictions. Although this quality improvement was temporary, it is an important result for planning to control environmental pollutants.

## Introduction

The coronavirus disease 2019 (COVID-19) pandemic caused by the SARS-CoV-2 virus emerged from Wuhan, China [1] and caused more than 250 million infected people and 5 million death up to 10 November 2020. COVID-19 is associated with multiple affecting factors such as air pollution, sociodemographic indices, which have a two-way relationship [2, 3]. After about 2 years, many countries especially developed countries with mass vaccination, have not yet been able to fully restore their social and economic activities to pre-2020 levels [4, 5]. The economic and social impacts have been significant and coinciding with the pandemic declaration of the disease by the World Health Organization (WHO) on 11 March 2020 [6], many countries put temporary closures of their industries and jobs on the agenda and on the other hand, restrictions within cities were seriously pursued in most countries [7]. These actions include restrictions on inter-city and intra-city traffic, closing businesses, closure of schools and small communities, suspension of tourist visas and so on. Most countries imposed national or local restrictions and closures until early May, after which the restrictions became easy and less severe [8, 9].

Considering the closure of industries and restrictions on community activities, including inter-city transportation and mobility, it is expected that these measures will have significant effects on the amount of air pollutants from industries and vehicles, especially in large cities [10, 11]. Important environmental pollutants include particulate matters (different combinations of solid, liquid and vapor particles), which are mainly in two types of particles with an aerodynamic diameter < 2.5 µm (PM_2.5_) and < 10 µm (PM_10_), tropospheric ozone [O_3_], nitrogen dioxide [NO_2_], sulfur dioxide [SO_2_], carbon monoxide (CO) [12]. The UK Department for Transport reported that traffic was significantly reduced (69%) during the holidays, which also had a significant impact on the concentration of environmental pollutants [13]. Nitrogen oxides are caused by the combustion of fossil fuels, which is a good indicator of vehicle-related air pollution. It has been shown that more than 50% and 23% of the total nitrogen oxides (NO_X_) in developed and developing countries are related to transportation [14]. This pollutant can also react with volatile organic compounds and the sun's ultraviolet rays to produce tropospheric ozone, which poses a serious health threat such as inducing airways inflammation and increasing airway hyperreactivity [15–17]. In previous studies, it was shown that particulate pollutants, e.g., PM_2.5_, PM_10_, NO_2_ and ozone were the most important pollutants contributing to COVID-19 mortality and incidence [18–20].

A report by the WHO states that about 7 million deaths worldwide every year are due to air pollution [21]. Also, according to the European Environmental Agency reports, 251,000–495,000, 31,000–76,000 and 9400–28,900 premature deaths are attributable to PM_2.5_, NO_2_ and O_3_ reported for the EU-28, respectively [22]. As such, according to the previous studies in India, about 1.24 million deaths are related to air pollution, of which 0.67 million are due to particulate pollutants [23, 24]. A study in China also estimated that reduction in NO_2_ leads to a reduction of about 8911 deaths [25]. In terms of total economic benefits of the protection of human life, nitrogen dioxide has had the greatest impact, with about $ 10 billion in about 20 major cities surveyed in the world [26]. Previous studies have shown that the highest incidence of air pollution-related diseases is in the Western Pacific and South-East Asia regions, which is associated with heavy industry and air pollution hotspots in these areas [27]. The effects of air pollution on different types of diseases have shown that these exposures play a significant role in the development and progression of a vast variety of diseases, especially in urban areas [24, 25, 28]. As a result of COVID-19, the widespread restrictions and closures in different countries were applied and, as a consequence, the results of studies in the United States [29], Italy [30], the United Kingdom [31], Spain [32], China [33] and India [34] have shown that the concentrations of indicator pollutants are significantly reduced [35], especially for nitrogen dioxide and particulate matter. This reduction in the concentration of pollutants improves the air quality index (AQI) of cities and has positive effects on community health. However, despite the widespread lockdown caused by COVID-19 in some areas, the concentrations of many pollutants, including sulfur dioxide and ozone, have increased [36–40].

So far, in most studies, only a limited number of cities in one or a few countries have been limitedly addressed, while the present study focuses on changes in the AQI of pollutants in important industrial and capital cities of the world before and after 2020 (and before and after mass vaccination programs). Inspecting changes in air pollutants can help to revise air quality guidelines in different countries and enable policymakers and researchers to examine the effects of reducing or increasing air pollution on the prevalence, incidence and burden of several diseases and then, if applicable, to apply preventive measures. On the other hand, the study of changes in AQI relative to their concentration can be interpreted in a more understandable way for the whole community, which is the focus of this study. Finally, the purpose of this study was to investigate the changes in the AQI related to standard pollutants in 87 industrial, densely populated and capital cities in different countries of the world before, during and after 2020.

## Methods

### Study area and data source

This is an ecological and city-level study based on the “Strengthening the Reporting of Observational Studies in Epidemiology” (STROBE) guideline for observational studies [41], which was designed to examine changes in air quality index (AQI) of various pollutants during the first 121 days of 2020 (due to the implementation of the most COVID-19 restrictions) and comparison with past and next year [40]. The inclusion criteria for the cities study is the World Air Quality Index (WAQI) project report from the website https://aqicn.org/; this report has been published online for employment of air pollution data on COVID-19 by researchers, during the first 6 months of 2015 to 2018 (January 1 to June 30) and full data for 2019 to 2020 has in more than 380 cities [42]. The distribution map of cities has already been published in a study by researchers [40].

**Fig. 1:**
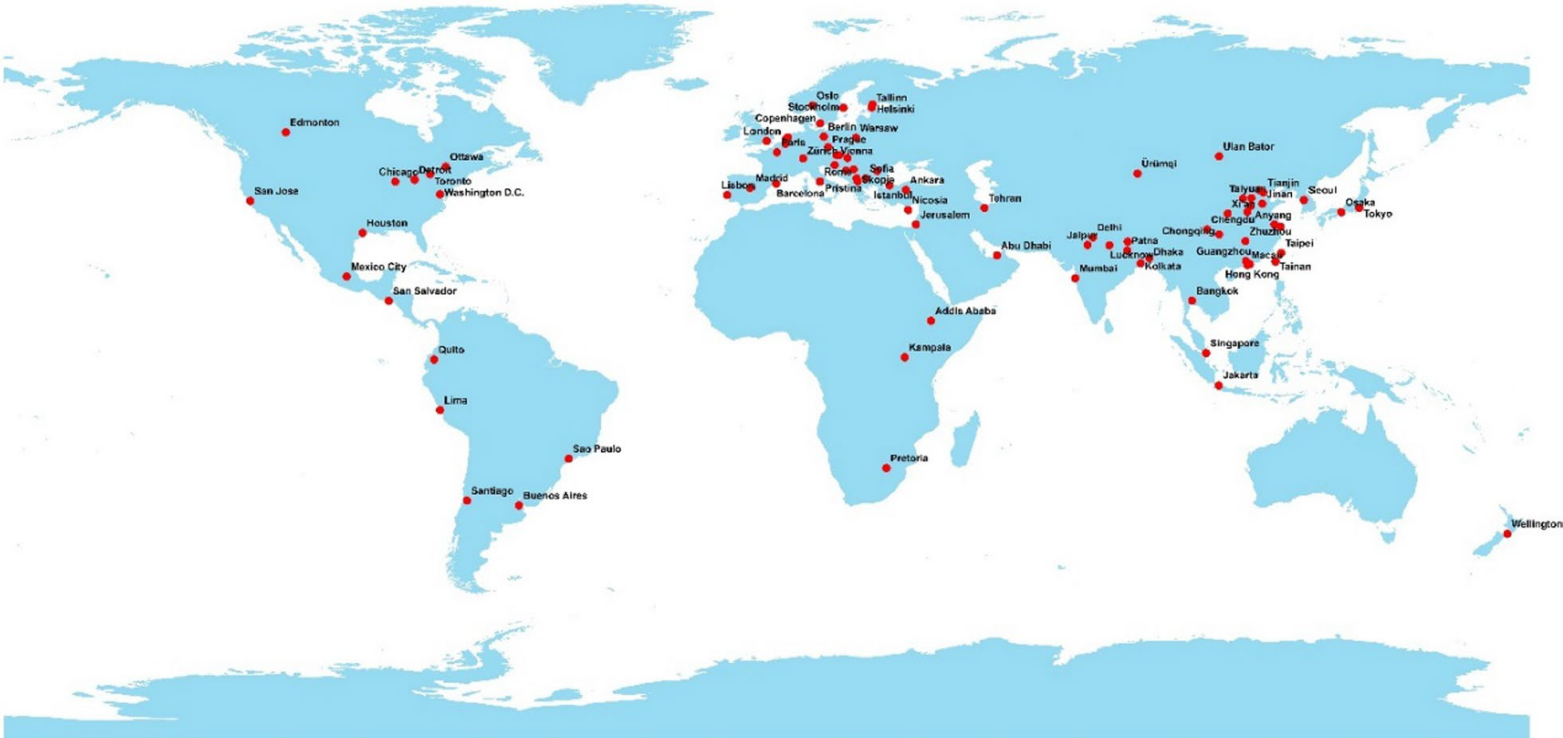
Map of world showing location in 87 different cities included in the study

Also, information about different cities from 2021 until April 30 has been published on the organization's website. According to the study objectives, cities that met the main inclusion criteria were included in the study. These criteria for selecting the cities in order of importance are as follows: (1) the AQI of different pollutants and meteorological variables for it should be fully published; (2) it should be the capital of the country and (3) It should be one of the top 100 polluted cities in the world (in terms of particles) according to the report of the World Health Organization https://www.who.int/. If in one country, the information for its capital was not complete, other cities of that country that had the third criterion were used. The choice of a country’s capital and major cities can be important because of the significance of maximizing the lockdown executive restrictions of countries. Latitude and longitude of cities were extracted from Google and the distribution map of cities was drawn in ArcGIS 10.3 software (Fig. 1). Due to the distribution of air pollution monitoring stations and additional information, most of the selected cities are from Asia and Europe. Further information is available on the WAQI website.

### Outcome and quantitative variables

**Fig. 2:**
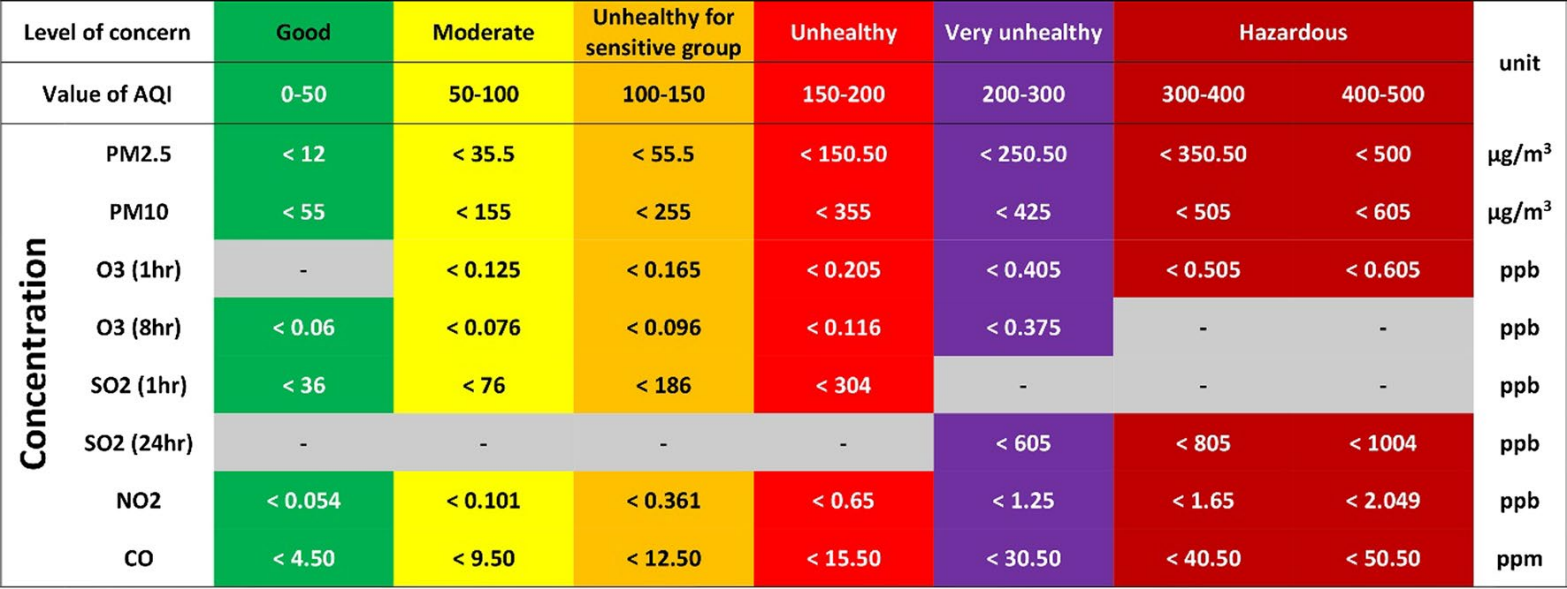
Air quality index and cut-off concentrations and health messages. Good air quality is considered satisfactory and air pollution poses little or no risk, moderate air quality is acceptable; however, for some pollutants there may be a moderate health concern, unhealthy for sensitive groups members of sensitive groups may experience health effects, unhealthy everyone may begin to experience health effects, very unhealthy health warnings of emergency conditions and hazardous health alert: everyone may experience more serious health effects

To analyze various air pollution indicators, we extracted the AQI values related to PM_2.5_, PM_10_, NO_2_ and SO_2_, CO and tropospheric O_3_ in the first 4 months of 2018 to 2021 and entered the Graph Pad Prism software. The pollutant concentrations were measured as ground-based measurements. The AQI calculation method for each pollutant was shown on the website https://aqicn.org/calculator. The values of the air quality index with respect to different pollutants along with their related color and description of the index are shown in Fig. 2.

The median (IQR = interquartile range), minimum and maximum daily AQI values were reported at different stations for the cities. The average values of the median for 1 month were considered as the monthly average. Then, the 4-month average was analyzed to compare the mean of 2018 to 2021 with the 2020 baseline as a lockdown scale and the severe restriction of countries. To control the quality of the data, we removed values equal to zero and abnormal numbers such as more than 1000 from the data. Also, each city for which pollutant information was reported for < 25 days (< 80%) was excluded from the study. If the calculated final outcome values were abnormal, the quality of the data in that city was rechecked to ensure the accuracy of the information. The outcomes examined include the percentage of changes in AQI pollutants compared to 2020 and the average AQI pollutants for each city.

In this study, we tried to use the average values of each year as a scale of comparison with next years, which shows the impact of lockdown in 2020 (severe restrictions) and 2021 (easier restrictions or coinciding with mass vaccination) with previous years. To investigate the effect of the restrictions in 2020 and 2021, the percentage difference of AQI values between different years was calculated by the following formulas [43]:

1 $${\%\Delta \mathrm{AQI}}_{2020x}=\frac{({\mathrm{AQI}}_{2020x}-{\mathrm{AQI}}_{2019x or 2018x})}{{\mathrm{AQI}}_{2019x or 2018x}}\times 100,$$

2 $${\%\Delta \mathrm{AQI}}_{2021x}=\frac{({\mathrm{AQI}}_{2021x}-{\mathrm{AQI}}_{2020x})}{{\mathrm{AQI}}_{2020x}}\times 100.$$

%ΔAQI_2020*x*_ indicates the percentage of AQI changes compared to 2018 and 2019. AQI_2020x_ represents the average AQI of pollutant *x* in 2020 and AQI_2019x_ or AQI_2018x_ is the average AQI of pollutant *x* in 2019 or 2018. For the percentage difference between 2020 and 2021, the same variables are defined. In this case, if the percentage of positive difference is reported, it indicates an increase in the values of the AQI in 2020 compared to other years and if it is negative, it means a decrease in AQI. In order to reduce the bias of changes in air pollutants, climate variables that have a direct and indirect effect on the concentration of pollutants were extracted for each city.

### Statistical analysis

Descriptive data of the study were presented using the median (IQR), maximum, minimum and first and third quartile reports using box and whiskers diagrams. AQI of ambient pollutants variables (PM_2.5_, PM_10_, NO_2_, SO_2_, CO and tropospheric O_3_) was stratified by Human Development Index (HDI) and Gross National Income (GNI). The statistical significance of mean differences in AQI variables among countries (developed or developing) and (L&LM: low and lower-middle income: < 4045$, upper-middle income: 4046–12,535$ and high income: > 12,535) categories was assessed using independent-sample t-tests. The United Nations considers countries with HDI ≥ 0.788 as “developed” and any score below that as “developing” [44]. Median and IQR for AQIs were also calculated stratified by HDI and GNI categories. The average of AQI for each city was also mapped using GraphPad Prism software. The data have been analyzed with GraphPad Prism and SPSS software. We assessed the correlation between AQI variables and meteorological parameters using Pearson and Spearman rank correlation coefficients based on normality of data. In addition, the statistical significance of differences in AQI variables among years (2020–2018, 2020–2019, 2021–2020) and GDP (high and low) categories was assessed using paired-sample *t*-tests [10]. We used multivariable linear regression to analyze AQIs in combination with meteorological parameters to each other.

## Results

### Description analysis of AQI variation

**Fig. 3:**
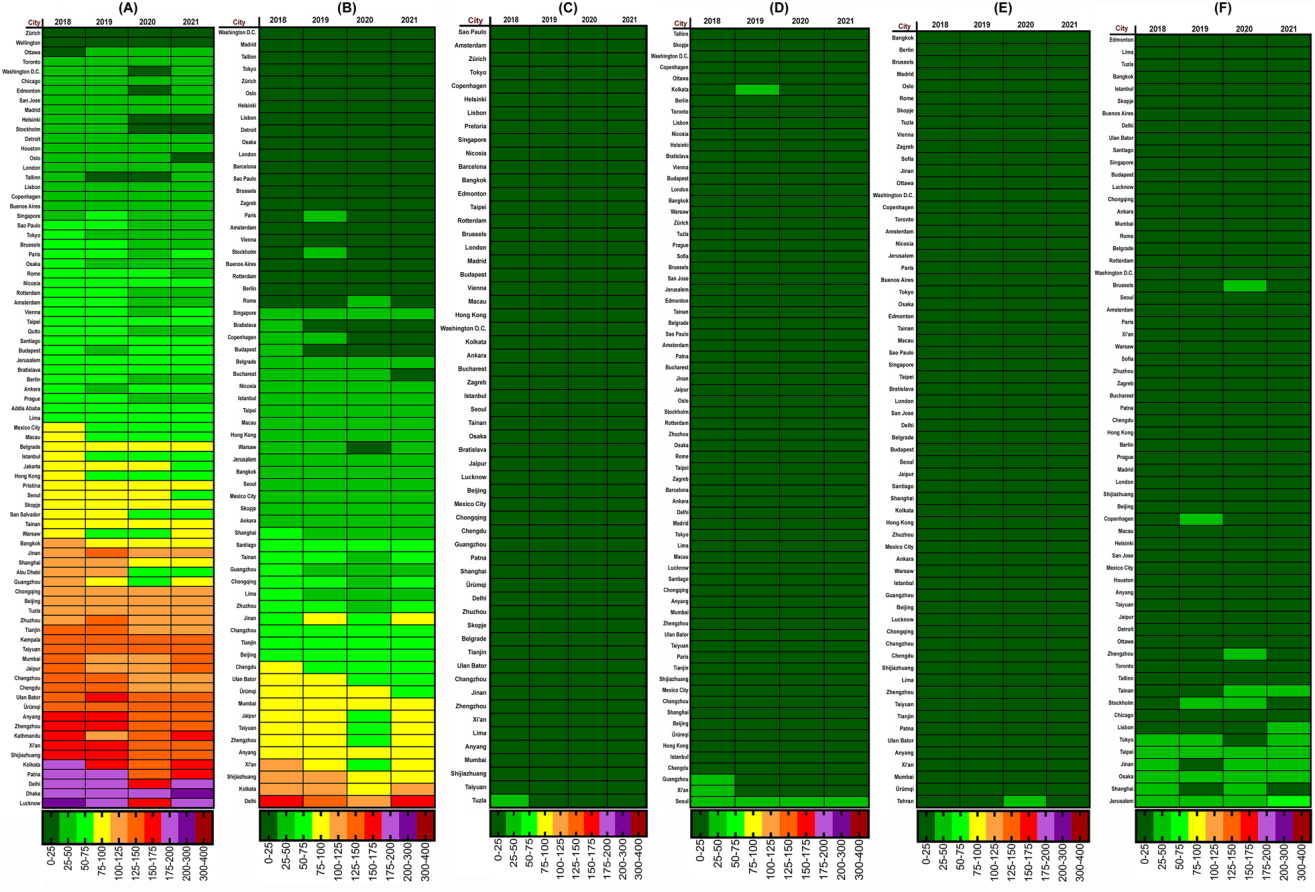
Heat Map created with GraphPad Prism showing average medians of the four first months the AQIs following 2020 lockdown and comparisons with 2018, 2019 and 2021. **A** AQI-PM_2.5_ variation, **B** AQI-PM_10_ variation, **C** AQI-SO_2_ variation, **D** AQI-NO_2_ variation, **E** AQI-CO variation and **F** AQI-O_3_ variation

A total of 87 industrial, polluted and crowded megacities from 57 different countries were included in the study. Of these, 58 cities are the main capitals of the countries and the rest are among the 100 most polluted and industrial cities in the world. Figure 3 shows the average AQI of various pollutants in cities with complete data in the first 4 months of 2018 to 2021 (January 1 to April 30). The results showed that Zurich (2.31), Tallinn (8.65), Washington (0.18), Tallinn (3.98) and Edmonton (0.10) had the lowest AQIs for PM_2.5_, PM_10_, SO_2_, NO_2_ and O_3_, respectively. During 2020, the cities of Dhaka (182.18), Delhi (106.36), Ulaanbaatar (11.19), Seoul (26.86) and Jerusalem (36.62) also had the highest values of AQI, respectively. In terms of AQI-CO, many cities had average values of 0.10 in 2020 and Istanbul with 19.69 had the highest AQI-CO.

**Fig. 4:**
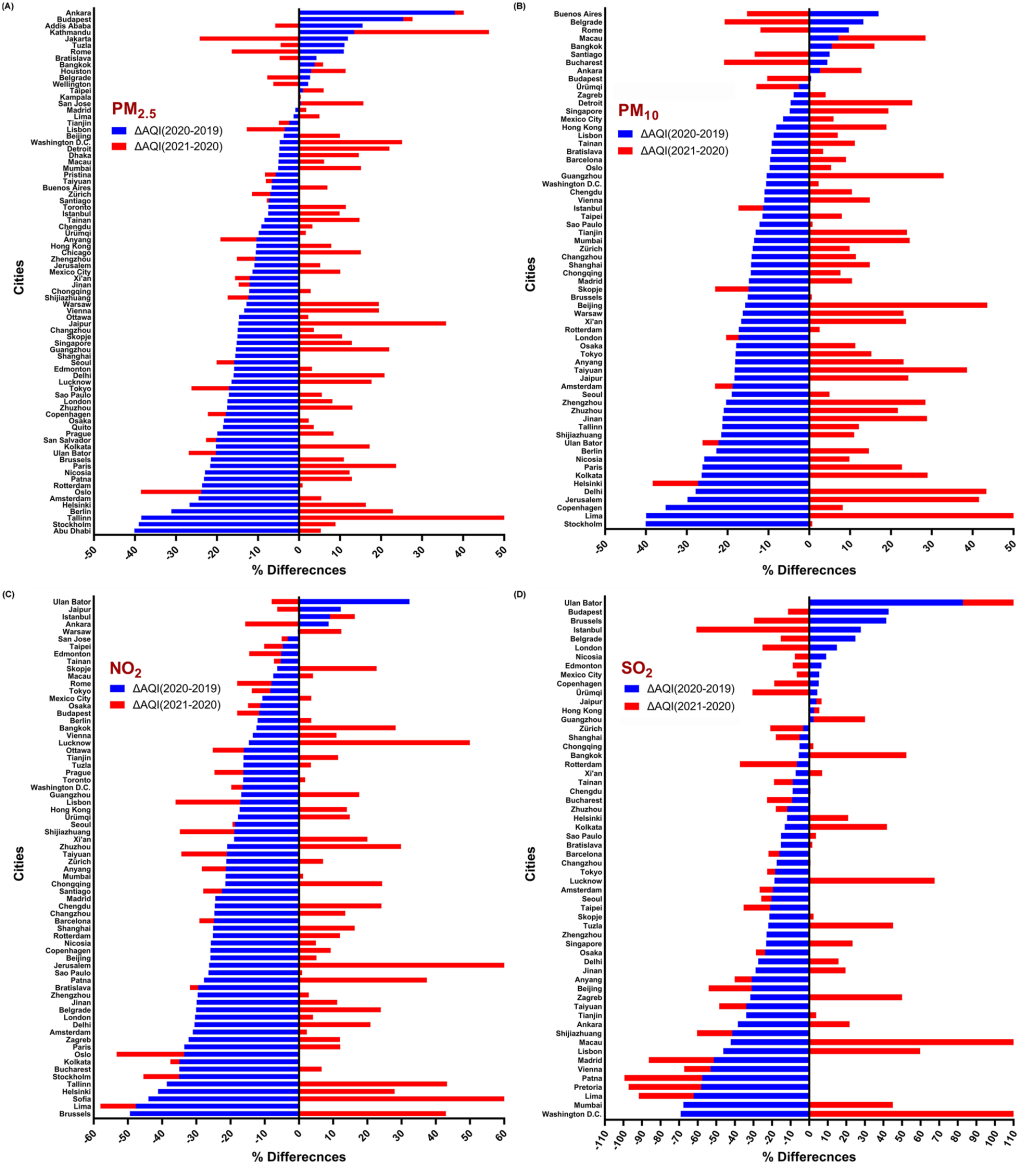
Percentage change of AQI PM_2.5_, PM_10_, NO_2_ and SO_2_ between 2020 during the lockdown period and 2019 and 2021 in different cities in the world. **A** AQI-PM_2.5_ variation, **B** AQI-PM_10_ variation, **C** AQI-NO_2_ variation and **D** AQI-SO_2_ variation

**Fig. 5:**
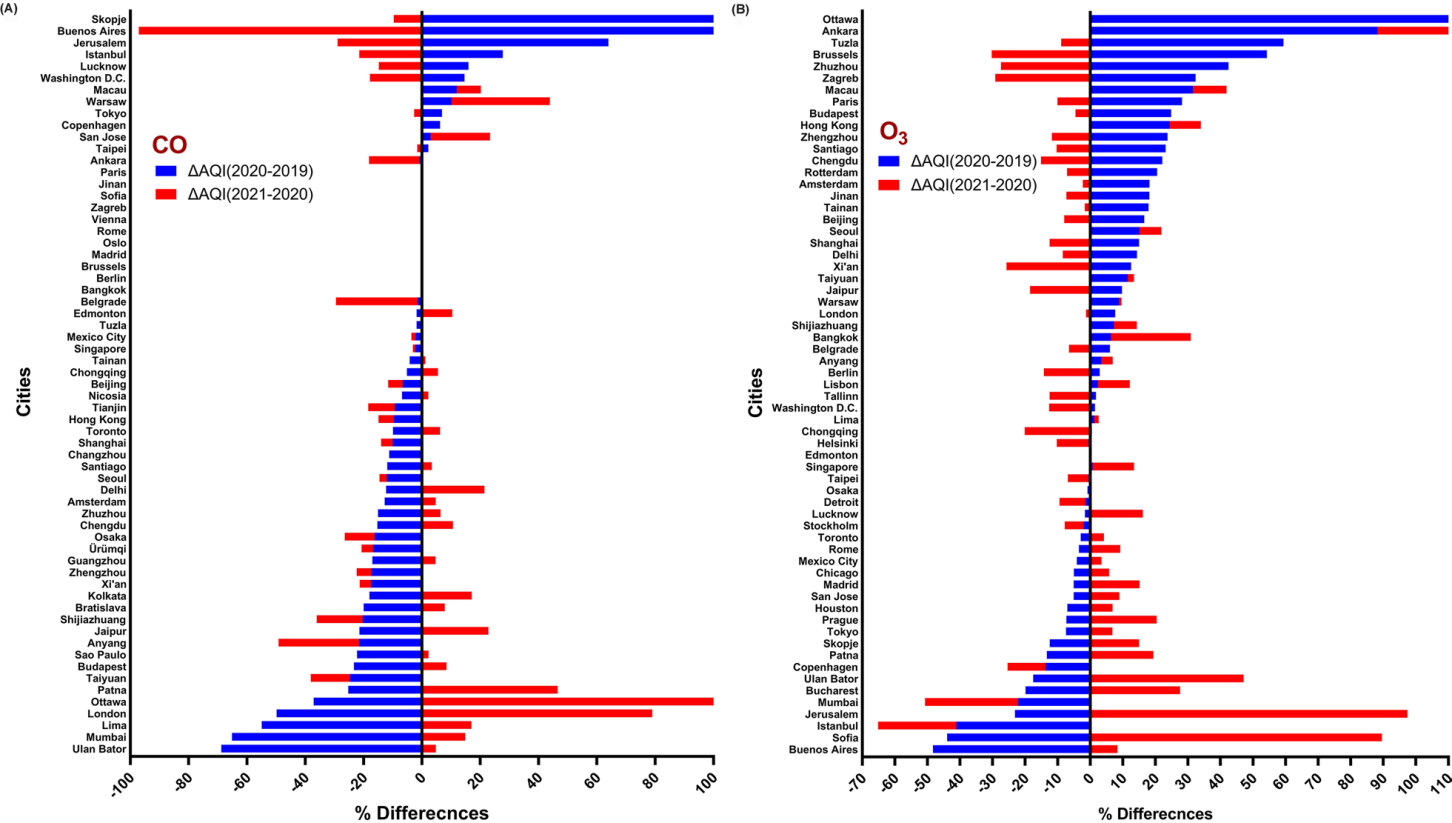
Percentage change of AQI-CO and O_3_ between 2020 during the lockdown period and 2019 and 2021 in different cities in the world. **A** AQI-CO variation and **B** AQI-O_3_ variation

According to the findings in Fig. 3, most of the problems caused by air pollution are related to particles, which are shown in Fig. 3A, B. AQI values in these pollutants have been reported for them up to 200–300 (Very Unhealthy). All cities surveyed had AQI < 100 for SO_2_, NO_2_, CO and O_3_. The results are shown in Figs. 4 and 5 to determine the percentage difference between the values of 2020 and 2019 and 2021 (before, during and after of lockdown).

According to Fig. 3, cities in which the quality index has decreased or increased, compared to 2019, could be distinguished. Based on Fig. 4, the cities of Abu Dhabi (− 40.13%) and Stockholm (− 40.05%) have the highest percentage decrease of PM_2.5_ and PM_10_ and the cities of Ankara (+ 37.97%) and Buenos Aires (+ 16.95%) have the highest percentage increase in 2020 compared to 2019.

Actually, in cities with a positive difference percentage, the AQI has not only decreased, but also increased. The results show that only 25% (17/67) and 13% (7/55) of the cities that had lower AQI-PM_2.5_ and AQI-PM_10_ in 2020 than in 2019 had also a decreasing trend in 2021, but in other cases, AQI values have increased during 2021.

The cities of Washington (-69.15%) and Ulaanbaatar (+ 82.78%) had the most negative and positive differences in terms of AQI-SO_2_ in 2020 compared to 2019, respectively, although air quality in Washington has declined in 2021 and has had the highest increase (+ 206.34%) among all cities.

Also, in terms of NO_2_ pollution, Brussels (− 49.41%) and Ulaanbaatar (+ 32.33%) reported the most negative and positive differences in 2020 compared to 2019, respectively.

**Fig. 6:**
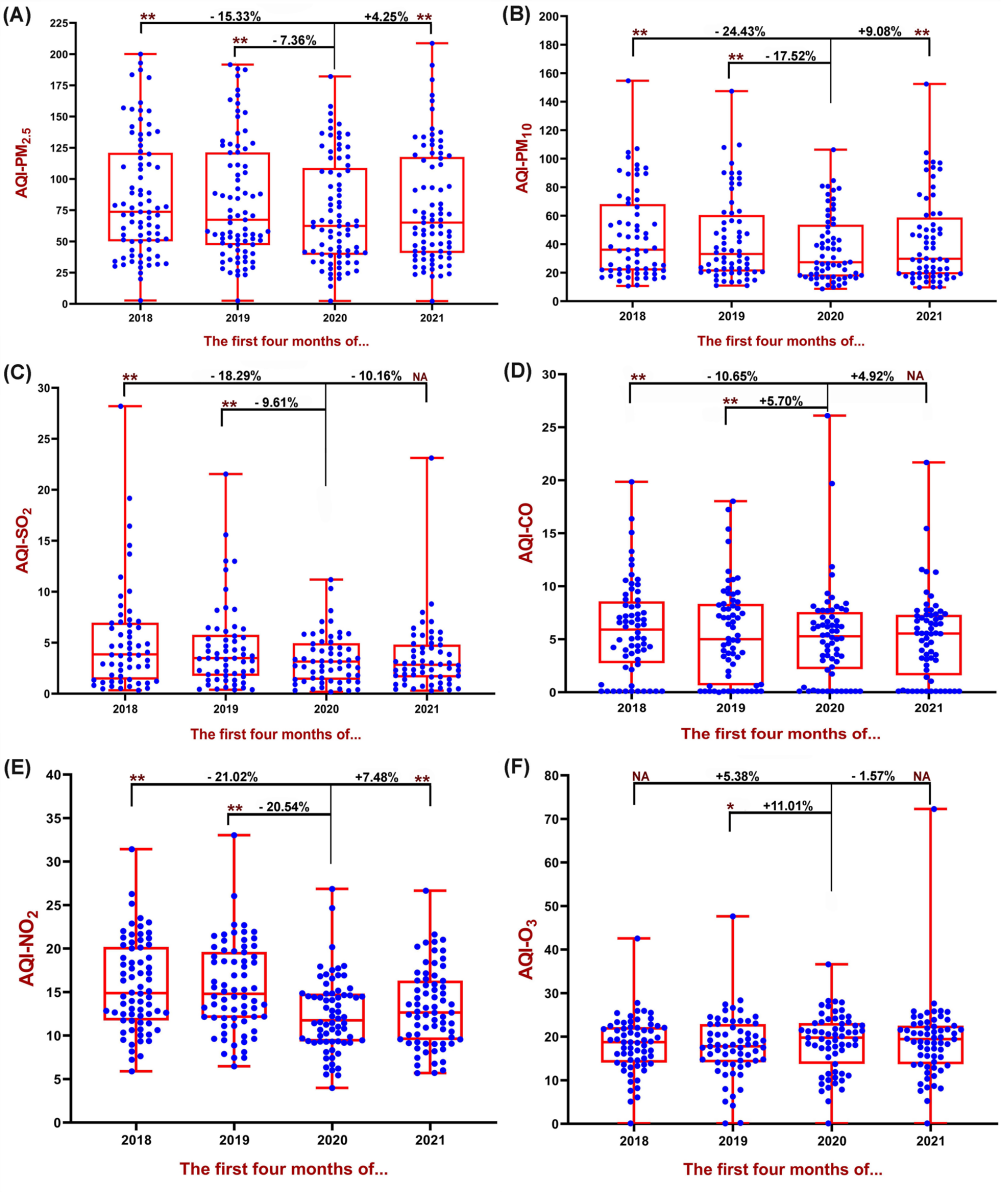
Box and whiskers plot depicting AQI variations for 87 cities in the World. The data show average of the first 4 months of 2018–2021 (January 1st–April 30th). **A** AQI-PM_2.5_ variation, **B** AQI-PM_10_ variation, **C** AQI-SO_2_ variation, **D** AQI-CO variation, **C** AQI-NO_2_ variation and **C** AQI-O_3_ variation. **P < 0.01, *P < 0.05, NA > 0.05

The result of AQI-CO illustrated that the cities of Ulaanbaatar (− 68.81%) and Skopje (+ 6089.22%) had the highest negative and positive differences compared to 2019, although in 2021 AQI-CO has significantly increased in the Ottawa (+ 124.64%). Another major pollutant in air quality is tropospheric O_3_, which has the highest percentage of negative and positive differences in 2020 compared to 2019 for the cities of Brussels (− 48.29%) and Ottawa (+ 9515.12%). The percentage difference of AQI for pollutants in other cities between 2020 with 2019 and 2021 can be seen in Figs. 4 and 5. The results showed that in general, AQI had a significant relationship between different pollutants in cities with complete data between 2020, 2018 and 2019 (*P* < 0.01), except for AQI-O3, which there was no statistically significant report (*P* > 0.05) between 2020 and 2018. Figure 6 (box and whiskers chart) shows the median values, along with the minimum, maximum and first and third quarters of the data for all cities. Also, the results of the Wilcoxon test analysis with star and NA symbols are shown in Fig. 5. It also reports the percentage difference between the median values of 2020 and 2018 as well as 2019 and 2021.

The results showed that the values of the AQI-PMs have decreased during rising the COVID-19 in 2020 (7.36% for PM_2.5_ and 17.52% for PM_10_), but during 2021, these values increased significantly (4.25% for PM_2.5_ and 9.08% for PM_10_). Also, AQI-SO2 has decreased compared to 2019 (9.61%), but for CO (5.7%) and O_3_ (11.01%), these values have increased. Also, the difference between the average AQI-NO_2_ has significantly decreased in 2020 compared to 2019 (20.54%) and 2018 (21.02%); however, these values have increased significantly in 2021 (7.48%).

**Fig. 7:**
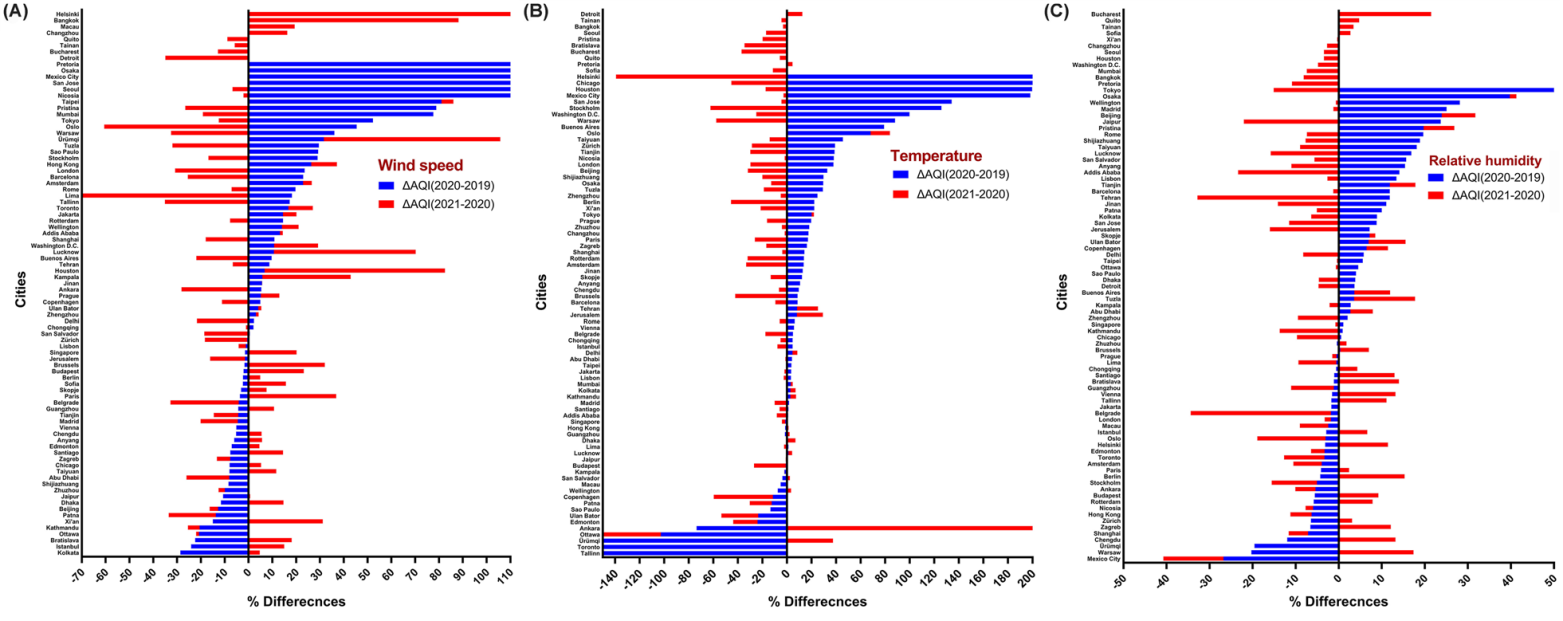
Percentage change of meteorological variables between 2020, during the lockdown period and 2019 and 2021 in different cities in the world. **A** Wind speed variation, **B** temperature variation and **C** relative humidity variation

Figure 7 is drawn to compare the percentage change of different cities for meteorological variables in 2020 compared to 2019 and 2021. Calcutta (− 28.59%), Tallinn (− 20,750.64%) and Mexico City ( −  26.77%) had the highest percentage of decrease in wind speed, temperature and relative humidity, while Pretoria (+ 1734.55%), Helsinki (+ 913.18%) and Tokyo (+ 85.37%) had the highest percentage increase in 2020 compared to 2019.

**Fig. 8:**
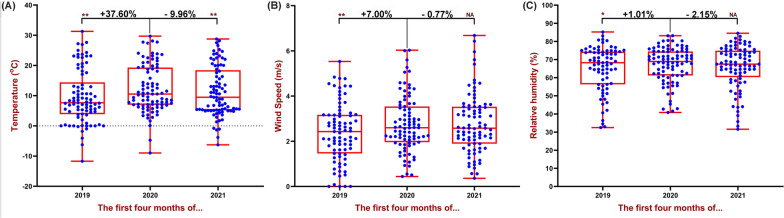
Box and whiskers plot depicting meteorological factors variations for 87 cities in the World. The data show average of the first 4 months of 2018–2021 (January 1st–April 30th). **A** temperature variation, **B** wind speed variation and **C** relative humidity variation. **P < 0.01, *P < 0.05, NA: *P* > 0.05

Figure 8 shows the box and whiskers diagrams for meteorological variables in the 3 years from 2019 to 2021. According to the findings of the study, the average temperature, wind speed and relative humidity in 2020 compared to 2019 have increased to values of + 37.60%, + 7.00% and + 1.01%, respectively (*P* < 0.05)

### Correlation and regression analysis of meteorological variables and AQI

**Fig. 9:**
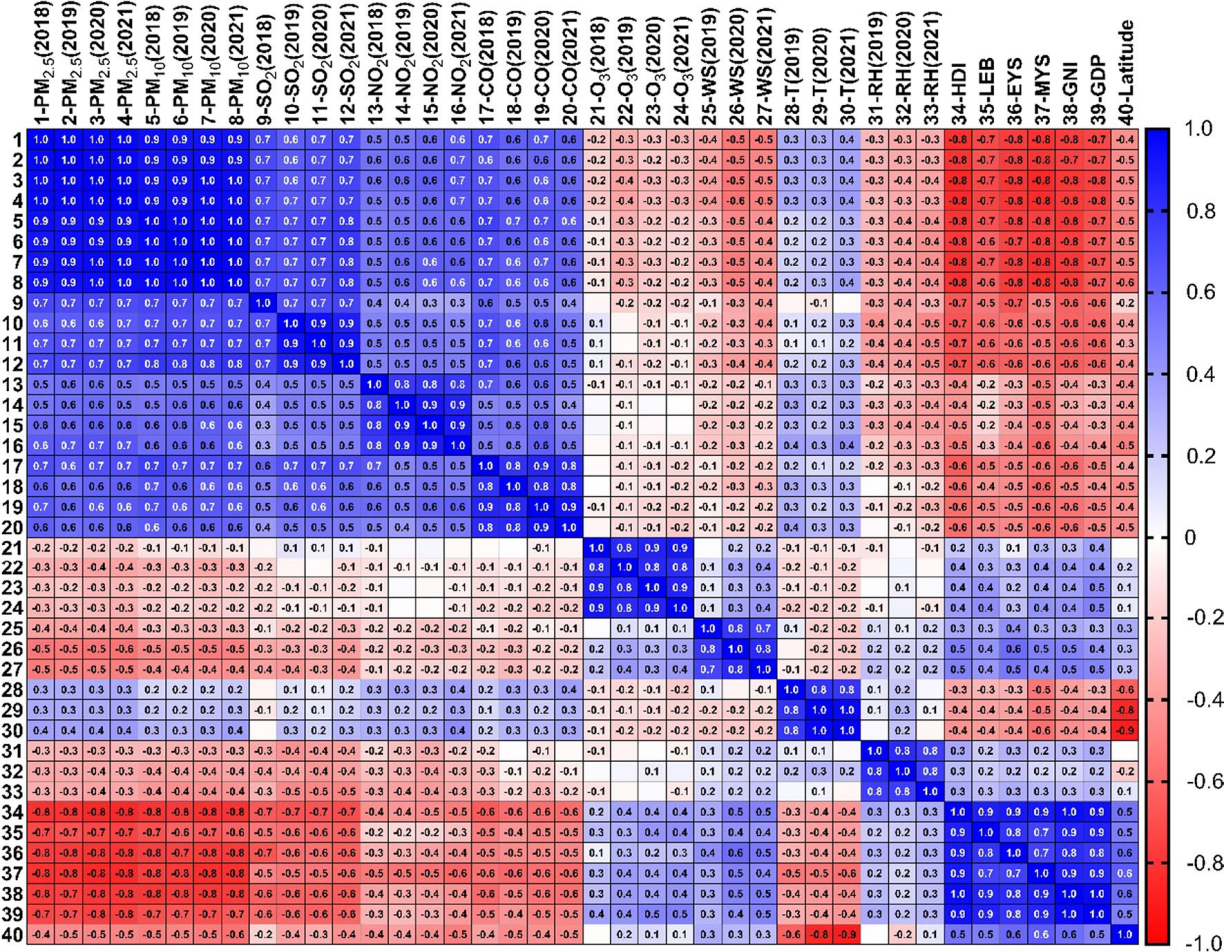
Correlations between AQI’s air pollutants and meteorological and socio-economic factors during 2020, lockdown periods and 2018, 2019 and 2021

The association between the AQI of different pollutants and meteorological variables for 2019, 2020 and 2021 is reported in Fig. 9.

The results of correlation analysis showed that there was a significant negative correlation between AQI-PMs and wind speed in all years (*P* < 0.05). However, there was a negative and significant correlation between wind speed and AQI-SO_2_ and AQI-NO_2_ only in 2020 (*P* < 0.05).

Also, a positive and significant correlation was observed between wind speed and AQI-O_3_ in 2020 and 2021 (*r* = 0.27 in 2020 and *r* = 0.39 in 2021; *P* < 0.05). AQI-PM_2.5_ and temperature also showed a positive and significant correlation in all 3 years (*P* < 0.01). Furthermore, a negative and significant difference was observed between relative humidity and AQI-PMs, as well as SO_2_ and NO_2_ in all years (*P* < 0.05).

The results of multiple regression analyses for the AQI of each pollutant by adjusting the meteorological variables are reported in Table 1. The results showed a correlation among AQI-PMs and AQI-SO_2_ with relative humidity (β = − 1.55 for PM_2.5_, β = − 0.88 for PM_10_ and β = − 0.10 for SO_2_, *P* < 0.001).

**Table 1:** Meteorological analysis of the relationship between factors associated with AQI parameters

Dependent AQI-variables	Wind speed (m/s)	Temperature (°C)	Relative humidity (%)
	β (SE)	β (SE)	β (SE)
AQI-PM_2.5_	− 3.75 (3.89)	0.59 (0.59)	− 1.55 (0.47)^**^
AQI-PM_10_	− 1.57 (2.49)	0.57 (0.37)	− 0.88 (0.30)^**^
AQI-SO_2_	− 0.07 (0.24)	< 0.01 (0.04)	− 0.10 (0.03)^**^
AQI-NO_2_	− 0.06 (0.46)	0.10 (0.07)	− 0.11 (0.06)
AQI-CO	0.96 (0.54)	0.13 (0.07)	− 0.08 (0.06)
AQI-O_3_	− 0.42 (0.75)	0.10 (0.12)	< 0.01 (0.09)

**P < 0.001

### Socioeconomic and dependent variables

**Fig. 10:**
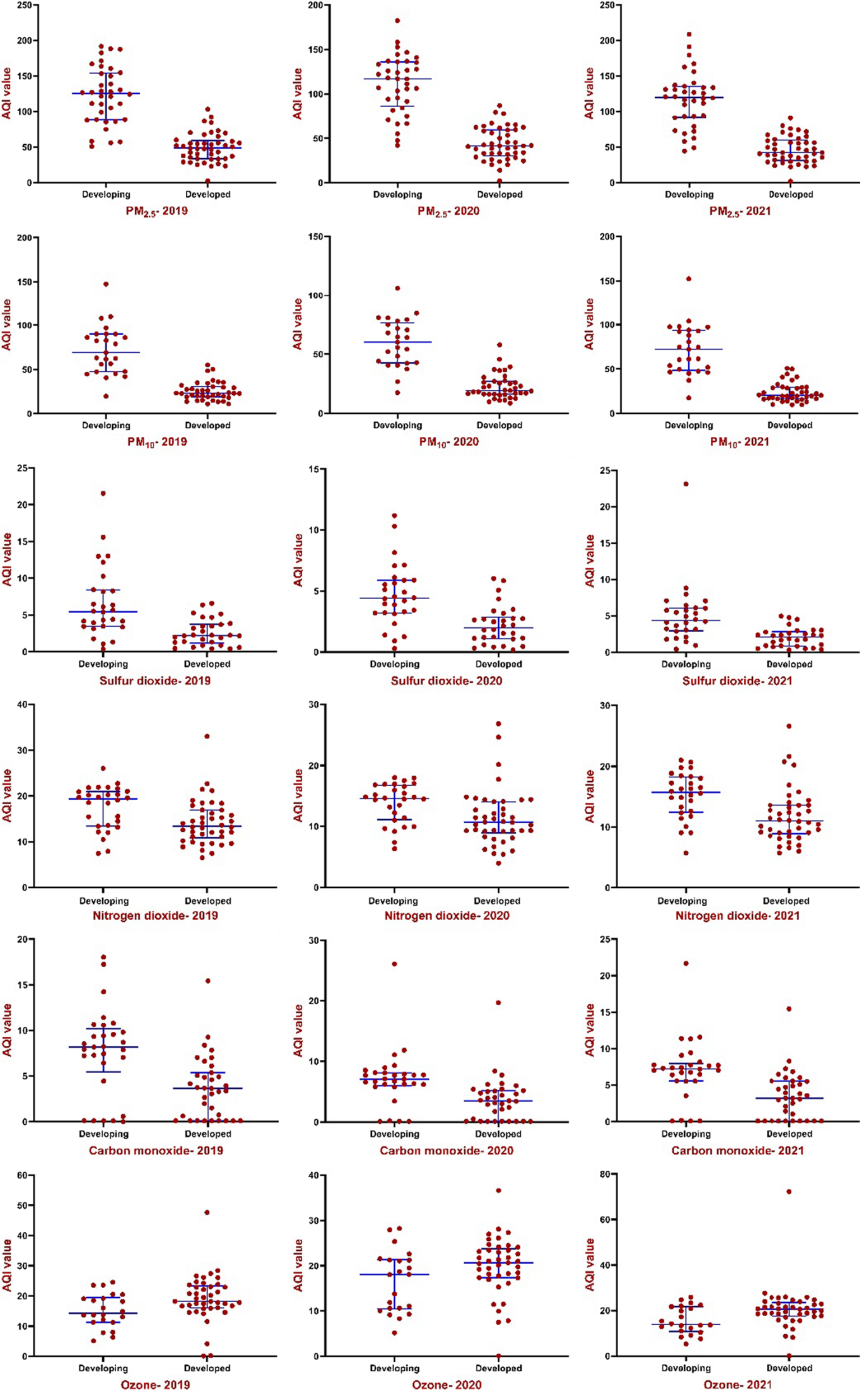
Differences of AQI parameters in developed and developing countries by independent-sample t-test. AQI values in developing countries were significantly higher than those in developed countries except for ozone. Horizontal blue lines represent group median with interquartile range

**Fig. 11:**
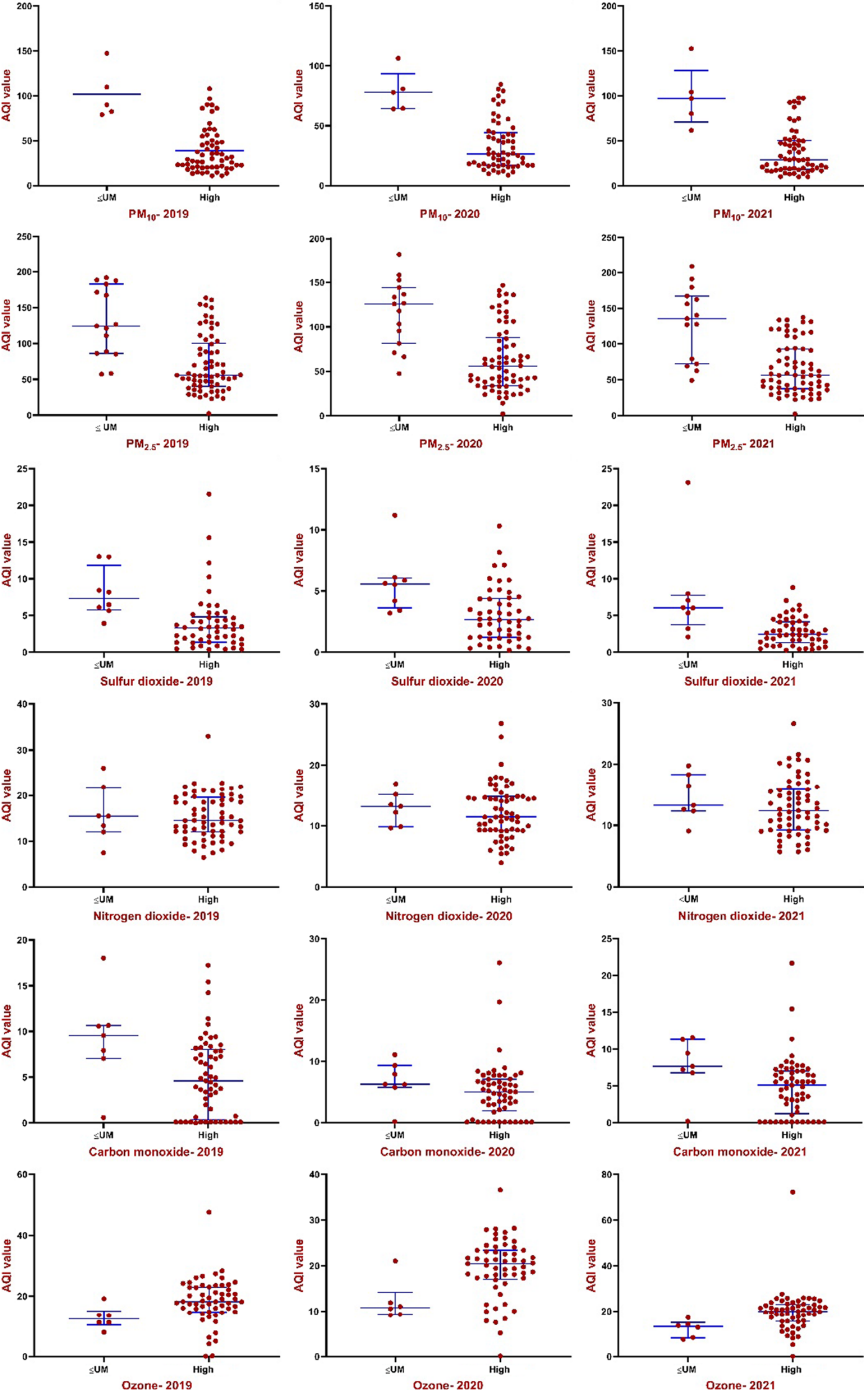
Differences of AQI parameters in high-income and upper-middle and lower (< UM) income countries by independent-sample *t*-test. AQI values in high-income countries were significantly lower than those in < UM countries except ozone. Horizontal blue lines represent group median with interquartile range

Other analyses were accomplished for country categories (based on HDI and GNI) to investigate the average AQI variables in different categories. There were significant differences between AQI parameters in developed and developing, as well as high-income and < UMI countries (*P* < 0.01) (Fig. 10). Among developing countries, the median (IQR) of AQI-PM_2.5_, AQI-PM_10_, AQI-SO_2_ and AQI-NO_2_ were 117.00 (IQR: 86.00–136.00), 60.20 (IQR: 42.60–76.50), 4.41 (IQR: 3.20–5.89), 14.60 (IQR: 11.10–16.80), which increased with decreasing national HDI (*P* < 0.01). Also, the mentioned AQI variables in high-income countries were lower than upper-middle income countries and increased with the decrease in national HDI (Figs. 10, 11). Furthermore, a negative and significant correlation was observed between HDI, GDP and GNI with AQI-PMs and AQI-CO (*r* > 0.65 for PMs and *r* > 0.40 for CO; *P* < 0.01). On the other hand, there is a positive association between HDI, GDP and GNI and AQI-SO_2,_ AQI-NO_2_ and AQI-O_3_ in all 3 years (*P* < 0.01). Furthermore, a negative and significant difference was observed between HDI, GDP and GNI, socio-economic variables and temperature, as well as a positive correlation for relative humidity and wind speed (*P* < 0.05). There is a positive association between wind speed and latitude, but a negative association for temperature and latitude (*P* < 0.01).

## Discussion

The results of the present study showed that the AQI of the first 4 months of the year in most cities in 2020, compared to the period before the COVID-19 pandemic (2019), has significantly improved; however, this has been reversed for many cities in 2021 (Figs. 4, 5). What is certain is that most large and industrial cities are exposed to particulate pollutants and nitrogen oxides [45], which according to the findings of the present study, the AQI-PM_2.5_ and NO_2_ during the lockdown period in 2020 have increased significantly (Fig. 6). These results belong to the outdoor environment, but indoor environments may be different [46–48]. Our findings are in line with several published studies that were indicative of reducing these pollutants due to lockdown and widespread restrictions in countries from the beginning of January to the end of May [11, 49–52]. Studies show that the Community Mobility Index, which represents the activities of educational sectors, transport, industries and social places, has significantly decreased during the first half of 2020 and has been effective in distributing concentrations of environmental pollutants [40]. Moreover, Fig. 3A clearly shows that in most of the cities surveyed, which were in poor condition in terms of AQI-PM_2.5_ in the same period before the pandemic, mentioned index was improved during the first 4 months of 2020 and with the establishment of COVID-19 restrictions and its related factors. Studies have shown that the use of e-Learning during the COVID-19 era might significantly reduce traffic and driving and thus reduce environmental pollution [53, 54].

The reduction of pollutants is also seen in Fig. 3B for PM_10_. Although PM_10_ concentrations in the environment are usually higher than PM_2.5_ but associated with PM_2.5_ pollution are greater in urban areas. Given the health problems caused by PM_2.5_, this improvement in the AQI will be noteworthy for health policymakers. The results showed that the highest levels of AQI-PM_2.5_ were observed in the cities of Patna, Delhi and Dhaka in 2019 and in the cities of Lucknow, Delhi, Dhaka, in 2020, respectively. Of the four cities mentioned, 3 are related to India and the other is related to Bangladesh, which are countries with low air quality and income. One of the reasons for the high number of PMs in these cities, in addition to the large transport fleet [55], is the use of household fuels such as wood and dry waste in these cities [10, 34, 56]. Also, in low-income or developing countries, due to the weak economy and lack of full implementation of environmental laws, the AQI in their cities is usually unfavorable [57, 58], which is in line with the results of our study.

India has been one of the countries with a sharp decline in the index of retail and recreation activities, transit stations and workplaces [40] and as a result, the sharp decline in AQI values in Indian cities may be due to this. It should be noted that to interpret the results of PM reduction, it is important to know the source of their production. PMs in an urban environment can be caused by the combustion of fossil fuels in vehicles as well as by emissions from various industries and natural events [59–62]. On the other hand, phenomena such as inversion in densely populated and industrial cities can act as a cover and prevent the spread of particles and pollutants in wider environments, thereby increasing the concentration of pollutants in the city. This phenomenon is more observed in areas with cold weather and cloudless skies, especially in winters [10, 63]. Overall, the findings showed that the average AQI-PM_2.5_ in 2020 compared to 2019 and 2018 was decreased by about 7% and 15% and AQI-PM_10_ declined by 18% and 24%, which indicates an overall improvement in PMs quality (Fig. 6A, B). It should be noted that many air quality monitoring stations may be located in areas close to major roads and airports, which will show high particulate matter levels.

Also, the average AQI-NO_2_ in 2020, compared to 2019 and 2018, has decreased by about 21% (Fig. 6E). The results of studies have shown that as the restrictions caused by COVID-19 in countries increased, the intensity reduction of PMs pollutants and NO_2_ have also increased [40, 64]. It has also shown that Gross Domestic Product (GDP) in most countries has declined during the second quarter of 2020, reflecting the lockdown impact on the economy and industrial closures [40]. Higher concentrations of pollutants are higher in developing cities except for ozone concentrations (Figs. 10, 11). Several studies showed that developing countries are in a poor situation in terms of air pollution than developed countries, which is due to strict environmental requirements and high-income status in developed countries [57, 65]. On the other hand, during the global economic recession from 2003 to 2007, it was shown that the concentration of NO_2_ emissions has decreased by about 20% [66].

Reports from European organizations revealed that the most important source of NO_2_ emissions in European countries is related to road transport [39, 67, 68]. It has also been reported that during the years from 2017 to 2018, the concentrations of NO_2_, SO_2_ and CO were decreased by 4.1, 6.7 and 4.3%, respectively [67]. In a study of 20 major cities around the world, Sannigrahi et al. reported that restrictions in countries between February 1 and May 11 have reduced the amount of NO_2_ by about 28 tons and CO by about 184 tons in these 20 cities [26]. On the other hand, it has reduced about 1310, 401 and 430 premature deaths associated with NO_2_, PM_2.5_ and PM_10_. However, previous studies have shown a significant association between air pollution and COVID-19 disease [69–71].

In general, AQI-SO_2_ has decreased by 11% in 2020 compared to 2019, which can be referred to as the closure of medium and large industries and travel restrictions in the transport fleet, which are the most important sources of sulfur production [72]. Also, the production of acid rain is of effects associated with high concentrations of SO_2_ and NO_2_; it has detrimental effects on the environment and aquatic ecosystems. In addition, acid rain on fertile lands washes away minerals and devalues soil [10, 73]. A study in Delhi, India, showed that lockdown significantly reduced the concentrations of SO_2_ and nitrogen oxide [74]. Also, the results of a study in New York showed that AQI-SO_2_ was decreased from 1.29 to 0.52 (59.68%) during the lockdown period and was decreased from 30.45 to 20.20 (33.66%) for NO_2_ [75].

Ozone levels compared to 2019 have generally increased by about 11% (− 48 to + 88%), which is consistent with various studies [36–38, 40]. Two studies in Barcelona, Spain [76], and Rajasthan, India [77], showed that, during the lockdown period, ozone concentrations were increased by about 50% and 45%, respectively. In addition, Wang et al. estimated that urban areas have significantly higher ozone levels than rural areas (145% vs. 46%) [76]. Studies have shown that NO_2_ is formed from the reaction of NO with ozone [78, 79] and if NO_2_ levels decrease, ozone levels are likely to increase, which an increase in AQI-O_3_ levels was observed in 60% of the cities of the present study, compared to 2019. Actually, due to the fact that ozone is a secondary pollutant and is dependent on precursors such as nitrogen oxides and volatile organic compounds (VOC), an increase in ozone concentration in the atmosphere can be expected if the emission of its precursors decreases [80].

Finally, it should be noted that meteorological variables can also, directly and indirectly, affect the concentration of pollutants in an area, which should be considered in interpreting the results of COVID-19 disease [3, 81]. Among the effects of meteorological variables are emission, transport, deformation and deposition [82]. The results of regression analyses showed that there is a significant inverse association between relative humidity and AQI-PM_2.5_, AQI-PM_10_ and AQI-SO_2_ in the presence of climatic variables of temperature and wind speed, while this association was reported to be positive and insignificant for ozone (Table 1). A study in three major cities in China has shown that the association between temperature and ozone is directly and the correlation between wind speed and air pollutants is the inverse [82], which is consistent with the results of our study. Another study in Turkey showed that low ambient temperature and low wind speed were associated with high concentrations of PMs and SO_2_ [83]. It is also commonly mentioned that ozone production can be limited in low temperature and high rainfall conditions [84].

## Strengths and limitation

To our best knowledge, this is among the first studies to examine the changes in AQI before, during and after 2020. In this study, considering the selection of large, industrial and capital cities as a sample size, the effects of lockdown and the executive constraints of countries can be further examined, compared and measured. On the other hand, according to the conducted literature review, most restrictions have been implemented in the first 4 months of 2020 and countries have reduced the severity of restrictions from May onwards; thus, in this study, the first 4 months in different years were intended as the basis of studies. In addition, the advantage of this study compared to other studies is the investigation of all indicators of standard pollution, which in the presence of each other, gives us a better view of the air quality of a city. However, the COVID-19 and environmental studies have several limitations that must be considered in each ecological study [85]. The first limitation is the location of air quality monitoring stations, which was not considered in this study due to lack of access. Also, the exact history of lockdowns is different for each city, which cannot be separated, but in general, considering the mean median of the data for several months and in different years, the impact of lockdowns with these differences is evident. This work did not take into account the specific situation of the epidemic situation of COVID-19 in various countries, the strict degree of epidemic prevention measures taken by various countries and the differences in the level of industrialization, urbanization and modernization of the cities. The reported result of higher AQI in high-income countries might confound by having good record stations in precise estimates on AQI. On the other hand, the elimination of some pollutants and especially meteorological variables (not adjusted in regression analysis) due to the lack of complete reporting of their values in all stations is another limitation of this study. Furthermore, the lack of access to long-term information on pollutants in cities was led to the consideration of fewer cities and comparison only with 2018 and 2019. Other factors such as improvement in AQI criteria, access to technologies services and increased awareness of the disease may occur also in countries with upper-level income that do not consider in this study. Failure to consider factors such as the transfer of particulate matters from one area to another and the factors affecting them in studies of environmental pollutants, especially particulate matters, are considered design limitations.

## Conclusions

Although the COVID-19 pandemic had irreparable destructive effects on human societies, it has been able to improve the air quality of most areas with executive restrictions in different countries. Findings illustrated that about 83, 86, 91, 76, 79 and 41% of cities revealed a decrease in AQI values of PM_2.5_, PM_10_, NO_2_, SO_2_, CO and O_3_ in 2020 compared to 2019. Also, the results showed that AQI generally improved for all pollutants except carbon monoxide and ozone in 2020, compared to 2019, in the 87 cities (8–25%); however, changes in 2021 compared to 2020 have been reversed and AQI values for PM_2.5_, PM_10_, CO and NO_2_ pollutants have generally increased (4–7%) due to the reduction of countries restrictions. In general, the implementation of strict laws related to COVID-19 restrictions can show the executive power of countries in reducing pollutants in non-crisis situations. Although this quality improvement was temporary, it is an important result that health policymakers can use to improve environmental conditions and maintain human health.

## Data Availability

All data generated or analyzed during this study are included in this published article (“Methods” section) and publicly available dataset. Data related to the AQI variables available in https://aqicn.org/ website. Also, the datasets used and/or analyzed during the current study are available from the corresponding author on reasonable request.

## Abbreviations

AQI: Air quality index
CO: Carbon monoxide
COVID-19: Coronavirus disease 2019
GDP: Gross domestic product
IQR: Interquartile range
NO_2_: Nitrogen dioxide
O_3_: Ozone
PM: Particulate matter
SO_2_: Sulfur dioxide
STROBE: Strengthening the reporting of observational studies in epidemiology
SD: Standard deviation
WAQI: World Air Quality Index
SARS-CoV-2: Severe acute respiratory syndrome coronavirus 2
UK: United Kingdom
US: United States
USEPA: US Environmental Protection Agency
WHO: World Health Organization
